# Efficacy of endoscopic intermuscular dissection vs. endoscopic submucosal dissection in treating rectal neuroendocrine tumors < 10 mm

**DOI:** 10.1055/a-2641-5725

**Published:** 2025-08-06

**Authors:** Guang Yang, Jingsong Wang, Bo Li, Xiaolong Xian, Jianzhen Ren, Qiuping Qiu, Xiaoping Hong, Longbin Huang, Suhuan Liao, Silin Huang

**Affiliations:** 1 701237 Department of Gastroenterology, South China Hospital, Medical School, Shenzhen University No. 1, Fuxin Road, Longgang District, Shenzhen, P. R. China

**Keywords:** Endoscopy Upper GI Tract, Endoscopic resection (ESD, EMRc, ...), Endoscopy Lower GI Tract, Polyps / adenomas / ..., Endoscopic resection (polypectomy, ESD, EMRc, ...)

## Abstract

**Background and study aims:**

Rectal neuroendocrine tumors (r-NETs) exhibit significant heterogeneity and malignant potential. Currently, endoscopic resection is the preferred treatment for r-NETs < 10 mm. However, traditional endoscopic resection carries a risk of positive vertical margins. This study aimed to compare clinical efficacy of endoscopic intermuscular dissection (EID) and endoscopic submucosal dissection (ESD) in treating small r-NETs (< 10 mm).

**Patients and methods:**

This retrospective study included 56 patients with r-NETs < 10 mm who underwent endoscopic treatment between April 2017 and September 2024 at Shenzhen University Affiliated South China Hospital and Shenzhen Hospital of Southern Medical University. All procedures were performed by the same surgeon. Patients were divided into two groups based on type of endoscopic treatment: the EID group (n = 16) and the ESD group (n = 40). We compared operative time, technical success rates, resection outcomes, adverse event (AE) rates, and histopathological findings between the two groups.

**Results:**

Median lesion size in the EID group (7.5 mm) was significantly larger than in the ESD group (6.0 mm) ( *P* = 0.001). Although operative time in the EID group was longer (39 vs 28.5 minutes), the difference was not statistically significant ( *P* = 0.137). The complete resection rate was 100% in the EID group and 97.5% in the ESD group, with no statistically significant difference. There were no significant differences in general characteristics, technical success rates (100% vs 100%), or incidence of AEs (bleeding, perforation, infection) (0% vs 0%) between groups ( *P* > 0.05).

**Conclusions:**

Endoscopic intermuscular dissection offers a better option for preventing positive basal margins and demonstrates good safety and feasibility.

## Introduction

Neuroendocrine tumors (NETs) are a heterogeneous group of neoplasms originating from neuroendocrine cells, capable of producing bioactive amines and/or peptide hormones. NETs can arise in various parts of the body, with the gastrointestinal system being the most common site, particularly involving the stomach, intestines, and pancreas 1 . Among these, r-NETs are among the more frequent types of gastrointestinal NETs, accounting for approximately one-third of such tumors and ranking as the second most prevalent gastrointestinal NETs in China 2 . With increasing use of colonoscopic screening, the detection rate for r-NETs has significantly risen 3 4 .

R-NETs are typically small in size, and many patients present without obvious clinical symptoms due to the insidious onset of the disease. Most r-NETs exhibit low malignancy potential, with histopathological grading often classified as G1, and the tumors are predominantly detected at T1 or T2 stages. Early detection and treatment are critical, because these tumors pose a potential risk of malignant progression 5 . According to guidelines from the European Neuroendocrine Tumor Society (ENETS) and the Chinese Consensus on Gastroenteropancreatic Neuroendocrine Tumors 6 7 , endoscopic resection is recommended for r-NETs smaller than 1.0 cm and graded as G1 or G2. For lesions measuring 1.0 to 2.0 cm without regional lymph node metastasis, local treatment can also be considered, whereas radical surgery is advised for tumors larger than 2.0 cm or those graded as G3.

With advancements in endoscopic techniques, minimally invasive endoscopic resection has become an increasingly important approach for treating r-NETs. Common endoscopic resection methods include endoscopic mucosal resection (EMR), modified endoscopic mucosal resection (M-EMR), endoscopic submucosal dissection (ESD), endoscopic mucosal resection with a ligation device (EMR-L), endoscopic full-thickness resection, and endoscopic intermuscular dissection (EID) 8 9 10 . Each of these methods has its own specific indications, procedural complexity, and potential complications. EMR, as a traditional method, is widely used due to its simplicity and short procedure time, making it suitable for small, superficial lesions. However, the relatively shallow resection depth of EMR can sometimes fail to achieve complete removal, especially for larger or deeper lesions, leading to residual tissue or positive margins. ESD offers a higher rate of en bloc resection compared with EMR, but it also carries a higher risk of complications, such as bleeding or perforation, and still poses a risk of positive resection margins 11 .

In recent years, EID has garnered attention for its potential in treating rectal tumors, with studies both domestically and internationally reporting its safety and efficacy, showing promising clinical outcomes 12 13 . EID is particularly suitable for lesions located deeper in the muscularis where traditional EMR or ESD may be insufficient, providing a new minimally invasive treatment option. However, to date, no direct comparison of clinical outcomes between EID and ESD for treatment of r-NETs has been conducted. Therefore, this study aims to compare clinical outcomes of EID and ESD in treating r-NETs smaller than 10 mm to assess their clinical value and provide a basis for clinical decision-making.

## Patients and methods

### Patients and study design

This study collected clinical data fromf patients with r-NETs who were hospitalized in the Department of Gastroenterology at South China Hospital of Shenzhen University and Shenzhen Hospital of Southern Medical University, and who underwent endoscopic resection between April 2017 and September 2024. Patients meeting the following inclusion criteria were included: 1) tumor diameter < 10 mm; 2) lesion located in the mucosal or submucosal layer; 3) postoperative pathology confirmed neuroendocrine tumor; 4) solitary rectal lesion; and 5) underwent EID or ESD treatment. Exclusion criteria were 1) presence of distant metastasis; 2) severe cardiovascular or cerebrovascular diseases or coagulation disorders; 3) incomplete clinical data; 4) refusal or inability to cooperate with endoscopic treatment; 5) presence of other malignancies; and 6) final pathology excluded NET diagnosis. A total of 56 patients were included in this study and were divided into the ESD treatment group (40 patients) and the EID treatment group (16 patients), based on endoscopic treatment method. We collected clinical data including gender, age, treatment method, tumor diameter, pathologic tumor size, vertical margin distance, complete resection status, technical success rate, pathological grading, operation time, intraoperative bleeding, postoperative complications, length of hospital stay, and hospitalization costs. All patients signed informed consent forms for endoscopic surgery before treatment. This study was approved by the Medical Ethics Committee of South China Hospital of Shenzhen University and adheres to the Declaration of Helsinki.

### Endoscopic evaluation and medication

The endoscopic procedures were performed by the same experienced endoscopist, using a forward-viewing endoscope model EG-760R (Fujifilm, Japan). Treatment methods included two endoscopic resection techniques: EID and ESD.

### EID Procedure

**Fig. 1:**
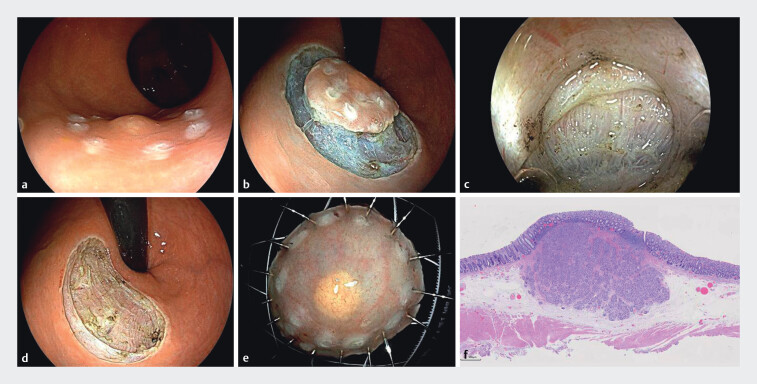
Endoscopic intermuscular dissection (EID). **a** Circumferential marking around the lesion. **b** Circumferential incision of the mucosa, exposing the muscularis propria. **c** Dissection between the circular and longitudinal muscle layers. **d** Post-dissection wound with intact longitudinal muscle. **e** Complete tumor resection. **f** Pathologically confirmed rectal neuroendocrine tumor.

First, a marking was made 5 mm around the lesion using a single-use mucosal incision knife (VDK-KM-20–165-D) with a high-frequency electrosurgical unit ( Fig. 1 ). A mixture of 0.9% sodium chloride solution and indigo carmine was injected into the submucosal layer using a single-use injection needle to ensure adequate lifting of the lesion. A circumferential incision was then made around the lesion, followed by gradual submucosal dissection until the muscularis propria was exposed. Dissection continued between the circular and longitudinal muscle layers of the muscularis propria, with any penetrating blood vessels encountered during the procedure being coagulated using the high-frequency electrosurgical unit for hemostasis. Finally, the lesion was completely resected, and exposed vessels were coagulated using single-use hot biopsy forceps. After thorough irrigation of the resection site, it was confirmed that there was no active bleeding, and the integrity of the longitudinal muscle layer was maintained, with no signs of perforation. The wound was then closed using a rotatable, reusable hemostatic clip (ROCC26195C, Micro-Tech, Nanjing). The resected specimen was flattened, fixed, and sent for histopathological examination.

### ESD Procedure

**Fig. 2:**
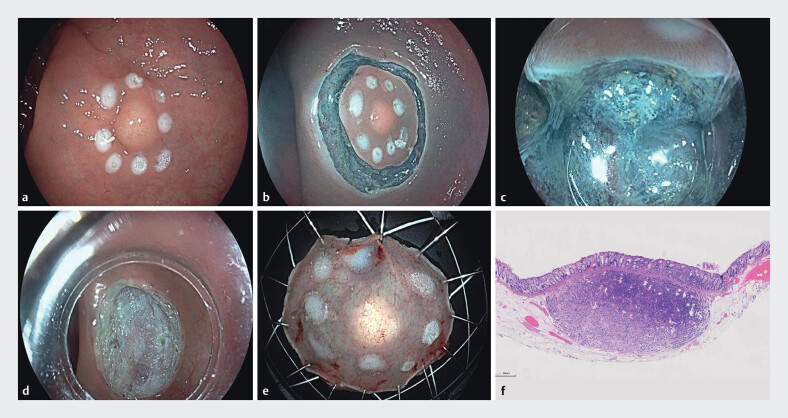
Endoscopic submucosal dissection (ESD). **a** Circumferential marking around the lesion. **b** Circumferential incision of the surrounding mucosa, exposing the muscularis propria. **c** Gradual dissection close to the muscularis propria. **d** Post-dissection wound with an intact muscularis propria. **e** Complete tumor resection. **f** Pathologically confirmed rectal neuroendocrine tumor.

The steps in ESD ( Fig. 2 ) were as follows First, the lesion was marked 5 mm around its perimeter using a single-use high-frequency electrosurgical knife. Then, a mixture of 0.9% sodium chloride solution and indigo carmine was injected into the submucosal layer using a single-use injection needle to ensure adequate lifting of the lesion. A circumferential incision was made around the lesion, and dissection was performed close to the muscular layer. The lesion was then completely resected. Exposed vessels were coagulated using a single-use high-frequency electrosurgical unit, and the resection site was checked to confirm no perforation. The area was thoroughly irrigated to ensure no active bleeding. After the procedure, the wound was closed using a single-use tissue clip, and the resected specimen was flattened, fixed, and sent for histopathological examination.

### Postoperative care and follow-up

After the procedure, all patients were required to fast and remain in bed while undergoing continuous monitoring of heart rate and blood pressure. Intravenous nutritional support was provided for 24 hours. During this period, patients were closely observed for signs of gas passage, bowel movements, bleeding, and any abdominal symptoms. If fever occurred, intravenous antibiotics were administered for anti-inflammatory treatment. Patients were advised to follow a low-residue diet and limit themselves to light physical activity for 1 week. All patients were pathologically confirmed, and endoscopic follow-up was scheduled annually for at least 5 years.

### Definitions

Complete resection rate (R0 resection rate) was defined as the percentage of patients who achieved en bloc resection with histopathologically confirmed negative horizontal and vertical margins among the total number of patients in the group who were pathologically diagnosed with rectal neuroendocrine tumors.

Technical success rate was defined as the proportion of patients in whom the tumor was successfully resected, regardless of the histopathological status of the margins. This metric reflects procedural success of the technique, indicating whether the tumor was completely removed during the procedure, without considering whether the margins were histologically negative.

Operation time was defined as duration from the initial marking of the lesion to the complete resection of the tumor. If additional endoscopic procedures were performed, the time for those procedures was excluded from the total operation time.

Postoperative complication rate was defined as the proportion of patients who developed complications within 14 days after surgery, among those pathologically confirmed with rectal neuroendocrine tumors. Complications included, but were not limited to, bleeding and perforation.

Intraoperative bleeding was defined as arterial spurting or persistent bleeding for more than 30 seconds during surgery, requiring the use of hemostatic forceps, vascular clips, or other endoscopic hemostasis techniques.

Postoperative delayed bleeding was defined as any of the following occurring between 48 hours and 14 days post-surgery: hematemesis, melena, dizziness, a drop in hemoglobin levels by 20 g/L from baseline, a blood pressure drop of more than 20 mm Hg, or an increase in heart rate by more than 20 beats/min.

Intraoperative perforation was defined as full-thickness damage to the rectal wall caused by the procedure, requiring repair with metal clips or other endoscopic techniques, or surgical intervention.

Postoperative perforation was defined as full-thickness damage to the rectal wall confirmed by colonoscopy or surgery, due to symptoms such as fever or peritonitis occurring postoperatively.

Surgical costs were the total costs of performing ESD or EID, excluding any additional endoscopic procedures performed concurrently.

### Outcomes and statistical analysis

Statistical analysis was performed using SPSS version 27.0. Continuous variables that followed a normal distribution were expressed as mean ± standard deviation, and comparisons were made using the independent sample *t* -test. Continuous variables that did not follow a normal distribution were presented as median (interquartile range), and comparisons were made using the non-parametric rank-sum test. Categorical variables were expressed as frequencies (percentages) and compared using the chi-square test or Fisher's exact test. *P* < 0.05 was considered statistically significant.

## Results

### Baseline data

In this study, the two groups of patients were similar in terms of gender and age, with no statistically significant differences ( *P* > 0.05) ( Table 1 ).

**Table:** **Table 1** Comparison of general characteristics between the two groups.

Characteristic	EID group (n = 16)	ESD group (n = 40)	Z/t/χ2	*P* value
Gender (male/female)	8/8	26/14	1.078	0.001
Median age (years)	45.56 ± 12.38	43.30 ± 10.40	-0.327	0.274
EID, endoscopic intermuscular dissection; ESD, endoscopic submucosal dissection.				

### Endoscopic findings

Endoscopic results showed that lesion size in the EID group was 7.5 cm (interquartile range [IQR] 6.0–8.5 cm), significantly larger than in the ESD group, which was 6.0 cm (IQR 5.0–7.5 cm), with a statistically significant difference ( *P* = 0.001). Although the operation time was longer in the EID group (39 minutes vs. 28.5 minutes), this difference was not statistically significant ( *P* = 0.137) ( Table 2 ). Regarding intraoperative bleeding, median blood loss in the EID group was 2 mL, whereas it was 1 mL in the ESD group, with no statistically significant difference ( *P* = 0.695) ( Table 3 ). In terms of surgical costs, median cost in the EID group was 9074 RMB, which was significantly higher than the 8534 RMB in the ESD group ( *P* = 0.004). Despite the slightly longer operation time in the EID group, the difference was not statistically significant ( *P* = 0.137), indicating no significant difference in operation time between the two methods. In addition, no perforations occurred during the procedure in either group, and the technical success rate was 100% in both groups ( Table 2 ).

**Table:** **Table 2** Comparison of endoscopic and pathological findings between the two groups.

Characteristic	EID group (n = 16)	ESD group (n = 40)	Z/t/χ2	*P* value
Tumor maximum diameter (mm)	7.5 (6.0–8.5)	6.0 (5.0–7.5)	-2.565	0.001
Operation time (min)	39.0 (27.5–49.5)	28.5 (24.5–46.5)	-1.489	0.137
Surgical costs (RMB)	9074.0 (8750.0–9522.0)	8534.0 (8302.0–8982.0)	-2.901	0.004
Pathologic tumor size (mm)	4.96 ± 1.20	3.09 ± 1.22	-4.547	0.001
Vertical margin distance (um)	1479.61 ± 901.77	393.50 ± 331.21	-4.874	0.001
Tumor grade (G1)	18 (100%)	40 (100%)	-	-
Technical success rate (%)	18 (100%)	40 (100%)	-	-
Complete resection rate (%)	18 (100.00%)	39 (97.50%)	-	1
EID, endoscopic intermuscular dissection; ESD, endoscopic submucosal dissection.				

**Table:** **Table 3** Comparison of adverse events and postoperative conditions between the two groups.

Characteristic	EID group (n = 16)	ESD group (n = 40)	Z/t/χ2	*P* value
Intraoperative blood loss (ml)	2.0(1.0–2.0)	1.0(1.0–2.0)	-0.392	0.695
Intraoperative perforation	0(0)	0(0)	-	-
Postoperative fever	0(0)	0(0)	-	-
Postoperative bleeding	0(0)	0(0)	-	-
Postoperative hospital stay (days)	4.25 ± 0.45	3.75 ± 0.54	-2.999	0.003
EID, endoscopic intermuscular dissection; ESD, endoscopic submucosal dissection.				

### Pathological findings

Pathological analysis showed that all 16 patients in the EID group and 40 patients in the ESD group had G1-grade tumors, with no cases of G2 grade, indicating no significant difference in pathological grading between the two groups. The complete resection rate in the ESD group was 97.5%, with one patient (2.5%) having a positive vertical margin and thus not meeting the R0 resection criteria, although no local recurrence or distant metastasis was observed during follow-up. Pathologic tumor size was significantly larger in the EID group compared with the ESD group (4.96 mm vs 3.09 mm, *P* = 0.001), and vertical margin distance was also significantly greater in the EID group (1479.61 µm vs 393.5 µm, *P* = 0.001). In the EID group, all patients had negative margins, achieving a 100% complete resection rate. Although one patient in the ESD group did not achieve R0 resection, there was no statistically significant difference in the complete resection rates between the two groups ( *P* > 0.05) ( Table 2 ).

### Postoperative recovery

In terms of postoperative recovery, the average hospital stay in the EID group was 4.25 ± 0.45 days, significantly longer than the 3.75 ± 0.54 days in the ESD group, with a statistically significant difference ( *P* = 0.003). However, no postoperative complications, such as fever or bleeding, were observed in either group (0% vs. 0%).

## Discussion

R-NETs, originating from neuroendocrine cells, are primarily located in the submucosal layer of the gastrointestinal tract and classified into low-grade (G1) and high-grade (G2, G3) tumors based on differentiation and biological behavior. G1 tumors grow slowly with a better prognosis, whereas high-grade tumors are more aggressive and prone to metastasis, resulting in poorer prognosis 14 . Incidence of r-NETs has increased in recent years, likely due to advancements in endoscopic screening techniques, which often detect small r-NETs (< 20 mm) incidentally during routine screenings. This highlights the crucial role of endoscopy in early diagnosis 15 . R-NETs are often asymptomatic or present with nonspecific symptoms such as mild rectal bleeding or changes in bowel habits, making early diagnosis challenging 16 . Accurate diagnosis relies heavily on endoscopy and pathological evaluation. Endoscopically, r-NETs typically appear as raised, yellowish, round or ovoid lesions, although their appearance can sometimes mimic other types of tumors, complicating differential diagnosis 17 .

In terms of treatment, selecting the appropriate therapeutic approach is critical due to biological characteristics and diverse clinical presentations of r-NETs. For small r-NETs < 10 mm in diameter, endoscopic therapy has become the primary treatment option. Techniques such as EMR and ESD are minimally invasive, with less trauma and faster recovery, making them well-suited for early-detected r-NETs 6 7 18 . In addition, as endoscopic techniques continue to evolve, procedures like EMR-L and EID have been introduced into clinical practice, offering more treatment options for r-NETs 19 20 .

Recent studies by Ito et al. highlighted the utility of EMR-L and cap-assisted EMR for treating colorectal NETs, particularly their feasibility in outpatient settings and lower costs 21 . However, our study focused on EID and ESD due to their established advantages in achieving negative vertical margins and addressing deeper submucosal lesions. Although EMR-L/EMRC are cost-effective for superficial, small tumors (≤10 mm), they carry inherent risks of incomplete resection in larger or deeper lesions. In contrast, our EID cohort achieved a 100% complete resection rate by dissecting within the intermuscular plane, even for median tumor sizes of 7.5 mm, compared with 97.5% in the ESD group (one case with positive vertical margin).

EID is an emerging endoscopic technique, particularly useful for managing larger r-NETs that extend deeper into the submucosal layer. Unlike ESD, EID involves dissection within the intermuscular space between the tumor and the surrounding normal tissues, allowing for a more complete resection of deep-seated lesions while minimizing damage to the deeper layers of the bowel wall. The key advantage of EID lies in its ability to achieve a higher rate of complete resection, especially for larger lesions, while reducing risk of positive resection margins. Although EID requires a higher level of technical expertise, it offers greater clinical potential in resection of complex lesions. Recent studies, both domestic and international, have increasingly demonstrated the safety and feasibility of EID in treating r-NETs, further supporting its role as a valuable option for r-NET treatment 22 .

This study compared clinical data and endoscopic findings of patients with r-NETs treated with EID and conventional ESD. The results indicated that, although there were differences between the two techniques in certain aspects, both demonstrated favorable outcomes in treatment of r-NETs. In terms of endoscopic findings, lesion size in the EID group was significantly larger than in the ESD group, which may be attributed to the advantage of EID in handling larger lesions. EID, by dissecting within the intermuscular space between the tumor and normal tissue, allows for more thorough resection of deep-seated lesions, making it better suited for larger tumors.

Although the operation time in the EID group was slightly longer, the difference was not statistically significant, indicating that time required for the two techniques is comparable. In addition, the two groups showed no significant differences in intraoperative blood loss or perforation rates, with both achieving a 100% technical success rate, suggesting that both techniques are equally safe and feasible. Pathological results revealed that the majority of lesions in both groups were G1 grade, with no G2 cases reported, indicating no significant difference in pathological grading between the two techniques. Notably, the EID group demonstrated significantly larger pathologic tumor size and greater vertical margin distance compared with the ESD group, reflecting that EID is more applicable for lesions requiring wider resection margins or involving larger tumor dimensions.​ The complete resection rate was 100% in the EID group and 97.5% in the ESD group. Although the EID group showed a slight advantage in complete resection, the difference was not statistically significant, demonstrating that ESD can also achieve effective resection in most cases. However, it is worth noting that no cases of tumor involvement at the resection margin were observed in the EID group, whereas one case in the ESD group had a tumor close to the cauterized margin. Although this did not result in local recurrence or distant metastasis, it suggests that EID may have an advantage in ensuring negative resection margins. Theoretically, by dissecting between the circular and longitudinal muscle layers of the muscularis propria, EID can more thoroughly remove deep-seated lesions, reducing the likelihood of positive margins. In terms of surgical costs, the expenses in the EID group were significantly higher than in the ESD group, which is largely due to the need for more titanium clips and more complex wound management in EID procedures—an unavoidable aspect of the technique. Regarding postoperative recovery, although length of hospital stay was statistically longer in the EID group compared with the ESD group, the difference was less than 1 day. Furthermore, no postoperative complications such as fever or bleeding were observed in either group, indicating that both techniques perform well in terms of controlling postoperative complications. There were several limitations to this study. First, this was a single-center retrospective study, which may introduce selection bias due to the nature of the retrospective studies. Second, the single-center design and involvement of a single endoscopist limit generalizability of the findings and may reflect individual expertise rather than universal applicability. Third, the relatively small sample size, particularly in the EID group, reduces the statistical power and may not fully capture true differences between the groups. Therefore, larger prospective studies are needed to further evaluate the complete resection rate and postoperative complications of EID in the treatment of r-NETs.

## Conclusions

In conclusion, EID shows a proven advantage in ensuring negative vertical margins, especially in cases of larger or deeper lesions. Furthermore, EID demonstrates excellent safety and feasibility, making it a valuable treatment option for r-NETs.

### Data availability statement

The data sets generated and/or analyzed during this study are available from the corresponding author on reasonable request.

## Notes

Correction **Efficacy of endoscopic intermuscular dissection vs. endoscopic submucosal dissection in treating rectal neuroendocrine tumors < 10 mm** Guang Yang, Jingsong Wang, Bo Li et al. Endoscopy International Open 2025; 13: a26415725. DOI: 10.1055/a-2641-5725 In the above-mentioned article an author's affiliation was corrected. The corrected affiliation is as follows: Department of Gastroenterology, South China Hospital, Medical School, Shenzhen University No. 1, Fuxin Road, Longgang District, Shenzhen, P. R. China. This was corrected in the online version on 10.09.2025.
