# Correction: Efficacy of endoscopic intermuscular dissection vs. endoscopic submucosal dissection in treating rectal neuroendocrine tumors < 10 mm

**DOI:** 10.1055/a-2698-2640

**Published:** 2025-09-12

**Authors:** Guang Yang, Jingsong Wang, Bo Li, Xiaolong Xian, Jianzhen Ren, Qiuping Qiu, Xiaoping Hong, Longbin Huang, Suhuan Liao, Silin Huang

**Affiliations:** 1 701237 Department of Gastroenterology, South China Hospital, Medical School, Shenzhen University No. 1, Fuxin Road, Longgang District, Shenzhen, P. R. China



In the above-mentioned article an author's affiliation was corrected. The corrected affiliation is as follows: Department of Gastroenterology, South China Hospital, Medical School, Shenzhen University No. 1, Fuxin Road, Longgang District, Shenzhen, P. R. China. This was corrected in the online version on 10.09.2025.

